# Chronic Condition Clusters and Polypharmacy among Adults

**DOI:** 10.1155/2012/193168

**Published:** 2012-08-01

**Authors:** Ami Vyas, Xiaoyun Pan, Usha Sambamoorthi

**Affiliations:** ^1^Department of Pharmaceuticals Systems and Policy, Robert C. Byrd Health Sciences Center (North), West Virginia University, P.O. Box 9510, Morgantown, WV 26506-9510, USA; ^2^Department of Community Health and Preventive Medicine, Morehouse School of Medicine, Atlanta, GA 30310-1495, USA

## Abstract

*Objective*. The primary objective of the study was to estimate the rates of polypharmacy among individuals with multimorbidity defined as chronic condition clusters and examine their associations with polypharmacy. *Methods*. Cross-sectional analysis of 10,528 individuals of age above 21, with at least one physical condition in cardiometabolic (diabetes or heart disease or hypertension), musculoskeletal (arthritis or osteoporosis), and respiratory (chronic obstructive pulmonary disease (COPD) or asthma) clusters from the 2009 Medical Expenditure Panel Survey. Chi-square tests and logistic regressions were performed to analyze the association between polypharmacy and multimorbidity. *Results*. Polypharmacy rates varied from a low of 7.2% among those with respiratory cluster to a high of 64.1% among those with all three disease clusters. Among those with two or more disease clusters, the rates varied from 28.3% for musculoskeletal and respiratory clusters to 41.8% for those with cardiometabolic and respiratory clusters. Individual with cardiometabolic conditions alone or in combination with other disease clusters were more likely to have polypharmacy. Compared to those with musculoskeletal and respiratory conditions, those with cardiometabolic and respiratory conditions had 1.68 times higher likelihood of polypharmacy. *Conclusions*. Rates of polypharmacy differed by specific disease clusters. Individuals with cardiometabolic condition were particularly at high risk of polypharmacy, suggesting greater surveillance for adverse drug interaction in this group.

## 1. Introduction

Care of individuals with multimorbidity defined as the coexistence of two or more chronic conditions is an emerging area of research. Existing studies have reported negative effects of multimorbidity on a variety of health outcomes such as disability, functional status, health-related quality of life, healthcare expenditures, and survival [1–12]. Multimorbidity is also associated with healthcare utilization specifically an increased number of hospital admissions [11] and prescription medications among individuals with multiple chronic conditions [13, 14]. In a US study of the elderly, it was found that an overwhelming majority (73%) with three or more chronic conditions were using 5 or more prescription drugs [15]. In an Australian study of multimorbidity across all age groups, it was reported that those with multimorbidity were 7 times to 22 times as likely to use four or more prescription medications as those without multimorbidity [13].

Multiple medications use often results in harmful drug-drug interactions [16]. A study by Nolan and O'Malley suggested that individuals taking 10 or more medications had over a 90% probability of experiencing one or more clinically significant drug interactions [17]. Such drug interactions have severe consequences such as hospital readmissions [18]. There have been many studies on polypharmacy among the elderly [19]; however, studies with specific focus on the association between polypharmacy generally defined as concurrent use of multiple medications and multimorbidity are very limited. One Italian study [20] of hospitalized elderly patients reported significant associations between specific clusters of diseases and polypharmacy. In this study, elderly patients with diabetes and coronary heart disease and cerebrovascular diseases had greater likelihood of polypharmacy compared to those without diabetes and cerebrovascular diseases. The adjusted odds ratio (AOR) was 9.8 with 95% confidence interval of 1.3–72.2, suggesting that coronary heart disease may increase the likelihood of polypharmacy. However, this study was limited to hospital settings. Additionally, polypharmacy issues have only been examined in the elderly populations while an Australian study documented that multimorbidity was prevalent (4.4% for 20–39 years of age and 15.0% for 40–59 years of age) in the younger adults as well [13]. Therefore, it is necessary to examine the relationship between polypharmacy and multimorbidity across all age groups.

The primary objective of the current study was to estimate rates of polypharmacy among individuals living in the community with specific clusters of chronic conditions, using a nationally representative sample of households in the United States. We also examined the independent relationship between specific clusters of chronic conditions and polypharmacy within a multivariate framework after controlling for demographic, socioeconomic, access to care, health status, lifestyle risk factors, and outpatient visits that may be associated with polypharmacy. We hypothesize that the rates of polypharmacy will depend on the specific chronic condition cluster and multiple conditions may not necessarily lead to greater rates of polypharmacy compared to those with single clusters.

## 2. Methods

### 2.1. Study Design

We used a cross-sectional study design. Data were extracted from the Agency for Healthcare Research and Quality- (AHRQ-) sponsored Medical Expenditure Panel Survey (MEPS) which is a nationally representative household survey of the United States noninstitutionalized civilian population [21]. The MEPS Household Component (HC) collected information on demographic and socioeconomic characteristics of household members, medical conditions, and treatments for these conditions including prescription drug use [21] over five interview rounds. Data on prescription drug use were collected through pharmacy providers and self-reports. A sample of medical providers were contacted to gather information on dates of visit, diagnosis and procedure codes, charges, and payments and were typically used as an imputation source to supplement/replace the data on household reported expenditures [21]. Medical conditions file was used to derive chronic condition clusters. The MEPS captured chronic and acute conditions of a respondent in several ways. (1) A disease condition may be reported in the Priority Condition Enumeration section in which persons are asked if they have been diagnosed with specific conditions; (2) a disease condition may be reported by the respondent when they had a particular medical event (hospital stay, outpatient visit, emergency room visit, home health episode, prescribed medication purchase, or medical provider visit); (3) a disease condition may be reported as the reason for one or more episodes of disability days; (4) a disease condition may be reported by the household level respondent as a condition “bothering” the person during the reference period [21]. A study by Machlin et al. [22] on sensitivity of household reported medical conditions in the MEPS found that household reports tend to be accurate for many conditions. Information on medical conditions was recorded verbatim and verbatim text was translated into International Classification of Diseases, 9th Edition, Clinical Modification (ICD-9-CM) codes by experienced and professional coders (http://meps.ahrq.gov/mepsweb/data_stats/download_data/pufs/h128/h128doc.pdf). For privacy reasons, 5-digit ICD-9-CM codes were also grouped into clinical condition classification codes. MEPS provided the crosswalk between ICD-9-CM and clinical condition classification codes.

### 2.2. Data

For purposes of our study, data were drawn from multiple files of MEPS (2009 survey) such as household, medical conditions, prescribed medicines, and office-based medical provider visits and outpatients visits. Medical condition files were used to identify specific clusters of chronic conditions. Polypharmacy use was defined from prescription medication files. Total outpatient visits were computed from office-based and outpatient visits files. Other independent variables were derived from the full-year consolidate household file.

### 2.3. Study Sample

Our study sample comprised living individuals over 21 years of age as of the end of 2009, reported having had at least one chronic physical condition in the following clusters: cardiometabolic (consisting of diabetes or heart disease or hypertension), musculoskeletal (consisting of arthritis or osteoporosis), and respiratory (consisting of COPD or asthma). Our analytical sample consisted of 10,528 individuals representing approximately 102 million individuals of the United States civilian noninstitutionalized population. This sample comprised 33.3% of total individuals interviewed during year 2009.

### 2.4. Measures

#### 2.4.1. Dependent Variable: Polypharmacy

There is no consensus in defining polypharmacy. However, a systematic review of the literature [23] stated “use of six or more concomitant medications” as one of the most cited definitions of polypharmacy. Accordingly we classified polypharmacy variable into two categories: (1) 0 to 5 drugs and (2) polypharmacy (≥6 drugs). MEPS prescription medications file contains information on therapeutic classes of medications through linkage of Multum Lexicon database (http://www.multum.com/Lexicon.htm). The unique therapeutic class codes were used to identify the maximum number of classes of medications taken by individuals in any one of the five interview rounds. We defined polypharmacy as using at least six prescribed medications in any one of the five rounds of interviews during year 2009.

#### 2.4.2. Key Independent Variable: Multimorbidity Categories

 We identified multimorbidity categories by grouping clusters of diseases based on specific organ domains [24]. Such clustering of disease conditions based on synergism in treatment patterns and self-management approaches have been used in our prior study [25]. Using a similar approach [25], we grouped three clinically meaningful disease clusters among diabetes, heart disease, hypertension, COPD, asthma, arthritis, and osteoporosis. These conditions were chosen due to their high prevalence, cost, morbidity, and mortality (http://meps.ahrq.gov/mepsweb/data_files/publications/st167/stat167.shtml) [26]. The specific clusters were (1) cardiometabolic (diabetes or heart disease or hypertension), (2) musculoskeletal (arthritis or osteoporosis), and (3) respiratory (asthma or COPD). These clusters were pooled together to form the following seven mutually exclusive categories of multimorbidity: (1) cardiometabolic conditions only; (2) musculoskeletal conditions only, (3) respiratory conditions only, (4) cardiometabolic and musculoskeletal conditions, (5) cardiometabolic and respiratory conditions, (6) musculoskeletal and respiratory conditions, and (7) cardiometabolic, musculoskeletal, and respiratory conditions.

#### 2.4.3. Other Independent Variables

*Demographic* variables consisted of gender (women, men), race/ethnicity (white, African American, Latino, other), age (22–39, 40–49, 50–64, 65–69, 70–74, and 75 and older), and marital status (married, widowed, separated/divorced, never married). *Socioeconomic* variables consisted of education (less than high school, high school, above high school), area of residence (metro, nonmetro), and poverty status (poor, near poor, middle income, high income). *Access to care* was assessed using health insurance coverage (private, public, uninsured) and usual source of care (yes, no). *Health status* was measured with variables such as perceived physical and mental health status (excellent/very good, good, fair/poor). *Lifestyle risk* factors comprised of body mass index (BMI) (underweight/normal weight, overweight, obese), current smoking (yes/no), and physical activity (vigorous activity 3 days a week/other). We also included *total number of visits* to either office-based provider or outpatient hospital clinics as a measure of contact with the healthcare system.

### 2.5. Statistical Techniques

Chi-square tests were used to assess significant differences between the multimorbidity categories and polypharmacy. Unadjusted and multivariate logistic regressions were used to analyze the association between polypharmacy and multimorbidity categories and other independent variables. We also contrasted the AORs of polypharmacy for specific multimorbidity categories. For example, we compared the AORs of polypharmacy between cardiometabolic and musculoskeletal clusters and cardiometabolic and respiratory clusters. In all these regressions, “0–5 drugs” category was compared to “polypharmacy.” All analyses used primary sampling unit, strata, and weights provided in the MEPS to control for clustering and unequal probability design and were conducted in survey procedures using SAS 9.2 to handle study weights and clustering.

## 3. Findings

Table 1 characterizes the study sample by multimorbidity categories in our study sample above 21 years of age, alive, with at least one of the cardiometabolic, musculoskeletal, and respiratory conditions in year 2009. Thirty-four percent of our study sample had cardiometabolic conditions and 25% had both cardiometabolic and musculoskeletal disease clusters; 4% had both cardiometabolic and respiratory disease clusters. However, only 7% of the study sample had all the three, cardiometabolic, musculoskeletal, and respiratory disease clusters.

Table 2 summarizes number and weighted percentages of individuals with polypharmacy by selected characteristics. Women compared to men were significantly more likely to be on polypharmacy (OR = 1.41, 95% CI = 1.27–1.56). Individuals in older age groups 40–49, 50–64, 65–69, 70–74, and 75 and older were also significantly more likely to be on polypharmacy compared to individuals in the age group 22–39. The odds ratios ranged from 2.03 to 7.70. There was also a positive and significant association between total outpatient visit quartiles and polypharmacy. Individuals who had visits in the upper quartile (4th quartile) were 17 times as likely as those with visits in the 1st quartile (OR = 16.77; 95% CI = 12.5–22.4).

We present weighted percentage of individuals with polypharmacy among different multimorbidity categories in the left panel of Table 3. As seen from Table 3, the highest rates (64.1%) of polypharmacy were found in sample individuals with all three (cardiometabolic and respiratory and musculoskeletal) disease clusters. The next highest rates (41.2% and 41.8%) were observed among those with cardiometabolic and musculoskeletal disease clusters and among those with cardiometabolic and respiratory disease clusters. The lowest rates were found in those with only musculoskeletal (7.9%) and only respiratory clusters (7.2%).

 Unadjusted logistic regressions and multivariable logistic regressions were used to examine the association between chronic condition clusters and polypharmacy. Odds ratios (OR) and AORs with their 95% confidence intervals for polypharmacy are presented in Table 3. Compared to individuals with all the three disease clusters (cardiometabolic, musculoskeletal, and respiratory), those with either one or two disease clusters were significantly less likely to receive polypharmacy. The unadjusted odds ratios ranged from 0.04 among those with respiratory conditions only to 0.40 among those with cardiometabolic and respiratory disease clusters.

We also examined the differences in the likelihood of polypharmacy between different single condition clusters. Compared to individuals with cardiometabolic disease cluster only, those with musculoskeletal cluster only and respiratory cluster only had lower odds ratios of reporting polypharmacy (OR = 0.38 and OR = 0.35, resp.). On the other hand, there were no significant differences in ORs between individuals with musculoskeletal conditions only and respiratory conditions only (OR = 0.91, 95% CI = (0.59, 1.39)).

When examining the differences in the likelihood of polypharmacy by two disease clusters, we found that individuals with both cardiometabolic and musculoskeletal clusters were more likely to report polypharmacy compared to those with both musculoskeletal and respiratory clusters (OR = 1.77). Similarly, individuals with both cardiometabolic and respiratory clusters were more likely to report polypharmacy (OR = 1.82) as compared to those with both musculoskeletal and respiratory clusters. Individuals with cardiometabolic and respiratory clusters did not significantly differ in the OR of polypharmacy from those with cardiometabolic and musculoskeletal clusters (OR = 1.03, 95% CI = 0.80, 1.32).

Multivariable logistic regressions to assess the association between multimorbidity and polypharmacy that controlled for gender, age, race/ethnicity, marital status, education, poverty status, health insurance, usual source of care, perceived physical and mental health, smoking status, BMI, and exercise revealed similar findings. For example, compared to individuals with cardiometabolic disease cluster only, those with musculoskeletal cluster only (AOR = 0.38; 95% CI = 0.30–0.49) and respiratory cluster only had lower likelihood (AOR = 0.62; 95% CI = 0.40–0.96) of polypharmacy. However, individuals with respiratory conditions had higher likelihood of receiving polypharmacy as compared to individuals with musculoskeletal conditions only (AOR = 1.62, 95% CI = 1.01–2.60).

## 4. Discussion

Our study estimated the rates of polypharmacy by multimorbidity categories among individuals with specific disease clusters such as cardiometabolic, musculoskeletal, and respiratory conditions. In our sample multimorbidity was highly prevalent with 39% of our sample having two or more disease clusters. In our sample, of all living adults over age 21, the prevalence of polypharmacy was 26%. While this rate is within the reported range from 5% to 78% of polypharmacy among the elderly [19], we are not able to find other studies in individuals across all age groups supporting the prevalence rates from our study. 

We found that multimorbidity was associated with polypharmacy and rates of polypharmacy varied across multimorbidity categories. When examined by two disease clusters, those with cardiometabolic conditions had higher rates of polypharmacy as compared to those without cardiometabolic conditions. Even when examined by single disease clusters, those with cardiometabolic conditions were more likely to have polypharmacy compared those with other single clusters. This finding is consistent with an Italian study [20] of hospitalized elderly patients. In the US, during office visits cardiovascular medication classes were a consistent part of polypharmacy between 1990 and 2000 [27]. High rates of polypharmacy among individuals with cardiometabolic conditions are not surprising because of the use of many therapeutic categories such as beta-blockers, angiotensin converting enzyme inhibitors, calcium channel antagonists, antiarrhythmics, and lipid lowering drugs [28–30] for synergistic management of these conditions. Indeed, the mean number of prescribed medications was above 7 for those with diabetes and/or heart disease [20]. On the other hand, for example, in COPD, on average an individual used only 3.5 medications [31]. Our study findings suggest that providers need to routinely monitor individuals with cardiometabolic conditions for adverse drug events and drug-drug interactions. In addition, a new drug classification system based on pharmacokinetic profiles and interaction potential that is currently proposed to combat the harmful effects of multiple medications use [16, 32] may go a long way in reducing adverse outcomes due to drug interactions in this group. 

Although highest rates of polypharmacy were found among elderly over 65 years of age, younger individuals between 50 and 64 years of age were also significantly more likely to report polypharmacy as compared to individuals in the age group 22–39 years. The prevalence of multimorbidity (i.e., the presence of all three disease clusters) was similar between 65 and older (9.0%) and adults in the 50–64 age group (8.2%). These two findings taken together suggest that multimorbidity in 50–64 age group was similar to those of elderly and the relationship between multimorbidity and polypharmacy is similar across the two groups. Again our findings are consistent with the Australian study by Taylor et al. [13], which reported that multimorbidity was associated with polypharmacy in all the adult age groups.

An interesting study finding was the association between greater number of visits and presence of polypharmacy, suggesting that ambulatory care visits increased the likelihood of polypharmacy. This finding is supported by existing literature. In a literature review on polypharmacy in the elderly, it was reported that [19, 28] five or more visits to a primary care physician increased the risk of polypharmacy by fifteen times. Approximately 75% of all the visits to primary care physicians end with a written prescription [33]. It is also plausible that the risk of polypharmacy could be increased to treat the high symptom burden among those with multiple conditions. However, a prospective randomized study at a single urban general practice in Ireland suggested that a ten-minute review of medications by general practitioners showed that 70% of the patients had stopped at least one medication after review [34]. This finding suggests that a routine medication review by primary care physicians can be incorporated to reduce the risk of polypharmacy among those with multiple chronic conditions.

Individuals with multimorbidity often see multiple providers to manage their chronic conditions and also may have the increased likelihood of hospitalization [11]. Although managed care organizations in the United States attempt to provide coordinated care, the financial incentives of the fee-for-service system in the country do not encourage integrated and coordinated care between providers, specifically between primary and specialized care providers [35, 36] and care sites (hospital and outpatient settings). Care from different providers without coordination often involves handoff of care among healthcare providers and changes to the medication regimens [37, 38], ultimately resulting in polypharmacy. Reducing polypharmacy will require coordinated efforts by physicians, nurses, and pharmacists [38].

Our study has several advantages such as use of nationally representative sample of adults and a large sample size, which allowed examination of polypharmacy across all adult age groups. Our definition of multimorbidity was based on synergism in treatment patterns and self-management approaches among different disease clusters [39]. Our study also included a comprehensive list of independent variables that may be associated with polypharmacy. Some limitations of our study need to be taken into account while interpreting the findings. All measures in the study were self-reported and subject to recall bias. Our study is an observational study and may suffer from selection bias. Additionally, we included only individuals with any of seven chronic conditions. We also did not measure severity of chronic conditions which may necessitate multiple medications use. Polypharmacy was broadly defined as number of therapeutic classes and in many of the chronic conditions combination therapies have become the norm rather than an exception.

 Despite these limitations, our study added to the existing literature by defining multimorbidity in term of disease clusters rather than count of conditions and also highlighting the variation in polypharmacy across different types of disease clusters. Future research needs to examine specific drugs in those with polypharmacy and identify potential drug combinations that may cause adverse drug events and adverse health outcomes. In addition, our study highlighted the need for routine surveillance to monitor polypharmacy and its adverse consequences among individuals with cardiometabolic conditions.

## Figures and Tables

**Table 1:** Weighted percentages of chronic condition clusters by sample characteristics. Medical expenditure panel survey, 2009.

	CM only	Musc. only	Resp. only	CM and Musc.	CM and Resp.	Musc. and Resp.	CM, Musc, and Resp.	
All	**34.3**	**19.5**	**7.5**	**25.1**	**4.0**	**2.9**	**6.9**	
Gender								^ ∗∗∗^
Women	28.0	21.0	8.2	26.7	4.1	3.7	8.4	
Men	42.2	17.6	6.6	23.0	3.8	1.9	5.0	
Age in years								^ ∗∗∗^
22–39	24.7	33.1	24.8	7.8	2.8	4.7	2.0	
40–49	34.0	26.8	9.6	16.7	4.4	3.7	4.7	
50–64	37.8	18.5	3.5	25.3	3.8	2.7	8.2	
65–69	38.3	9.3	2.7	32.7	4.7	3.1	9.2	
70–74	36.8	11.2	2.2	35.2	4.3	1.2	9.0	
75 and older	32.9	9.3	1.6	42.0	4.5	0.9	8.8	
Race/ethnicity								^ ∗∗∗^
White	32.5	20.0	7.7	25.3	4.1	3.2	7.1	
African American	42.7	13.1	5.6	26.4	4.4	1.2	6.6	
Latino	36.0	21.3	7.8	23.1	3.6	2.5	5.8	
Other	38.9	21.5	7.4	21.8	2.8	1.8	5.8	
Health insurance								^ ∗∗∗^
Private	36.4	20.7	8.3	22.5	3.6	2.9	5.6	
Public	29.1	12.8	4.2	34.8	5.1	2.6	11.4	
Uninsured	33.1	28.0	10.6	17.0	3.5	3.5	4.3	
Poverty status								^ ∗∗∗^
Poor	27.0	18.8	7.3	26.9	4.9	3.9	11.3	
Near poor	31.3	16.6	6.2	28.6	5.2	3.1	9.1	
Middle income	34.0	19.4	8.4	25.3	3.6	2.8	6.5	
High income	38.1	21.0	7.4	22.7	3.4	2.5	4.9	
Total visits								^ ∗∗∗^
1st quartile	40.6	27.3	12.3	12.2	2.8	2.6	2.1	
2nd quartile	41.8	17.8	8.9	21.5	3.9	1.8	4.4	
3rd quartile	33.9	17.6	5.8	28.6	4.0	2.3	7.7	
4th quartile	24.0	17.6	4.6	33.2	4.8	4.4	11.3	

Note: Based on 10,528 adults alive at the end of 2009, reported having at least one chronic condition (cardio-metabolic, musculoskeletal, or respiratory condition). All analyses accounted for complex survey design of the Medical Expenditure Panel Survey. Asterisks represent statistically significant group differences based on chi-square tests. CM: Cardio-metabolic; Musc.: musculoskeletal; Resp.: respiratory.

***P < 0.001; **0.001 ≤ *P* < 0.01; *01 ≤ *P* < 0.05.

**Table 2:** Number and weighted percent with polypharmacy. Unadjusted odds ratios and 95% CI from logistic regression on polypharmacy. Medical Expenditure Panel Survey, 2009.

			Logistic regression		
	*N*	Wt %	OR	95% CI	Sig
All	**2,692**	**25.5**			
Gender					
Women	1,727	28.4	1.41	(1.27, 1.56)	^ ∗∗∗^
Men	965	22.0			
Age in Years					
22–39	162	8.4			
40–49	284	15.7	2.03	(1.58, 2.61)	^ ∗∗∗^
50–64	956	25.1	3.67	(2.93, 4.59)	^ ∗∗∗^
65–69	371	37.2	6.50	(5.14, 8.21)	^ ∗∗∗^
70–74	291	40.3	7.40	(5.73, 9.56)	^ ∗∗∗^
75 and older	628	41.3	7.70	(6.17, 9.60)	^ ∗∗∗^
Race/ethnicity					
White	1,587	27.2			
African american	556	23.9	0.84	(0.73, 0.97)	^ ∗^
Latino	383	18.4	0.60	(0.51, 0.72)	^ ∗∗∗^
Other	166	19.4	0.65	(0.51, 0.82)	^ ∗∗^
Health insurance					
Private	1,336	22.4			
Public	1,231	39.5	2.26	(2.01, 2.55)	^ ∗∗∗^
Uninsured	125	10.6	0.41	(0.33, 0.52)	^ ∗∗∗^
Poverty status					
Poor	557	30.3	1.53	(1.33, 1.76)	^ ∗∗∗^
Near poor	691	31.4	1.62	(1.40, 1.86)	^ ∗∗∗^
Middle income	741	24.7	1.16	(1.02, 1.32)	^ ∗^
High income	703	22.1			
Total visits					
1st quartile	113	5.1			
2nd quartile	433	13.8	2.95	(2.18, 3.99)	^ ∗∗∗^
3rd quartile	778	27.1	6.88	(5.17, 9.15)	^ ∗∗∗^
4th quartile	1,368	47.6	16.77	(12.5, 22.4)	^ ∗∗∗^

Note: Based on 10,528 adults alive at the end of 2009, reported having at least one chronic condition (cardio-metabolic, musculoskeletal, or respiratory condition). All analyses accounted for complex survey design of the Medical Expenditure Panel Survey. Asterisks represent statistically significant group differences based on chi-square tests. CM: Cardio-metabolic; Musc.: musculoskeletal; Resp.: respiratory; Wt: weighted; OR: odds Ratio; CI: confidence Interval.

***P < 0.001; **0.001 ≤ *P* < 0.01; *0.01 ≤ *P* < 0.05.

**Table 3:** Weighted percentage with polypharmacy. Unadjusted and adjusted odds ratio and 95% confidence intervals for chronic condition clusters. From logistic regressions on polypharmacy. Medical Expenditure Panel Survey, 2009.

	Weighted percentage with polypharmacy	Estimates from logistic regressions on polypharmacy					
	Percentage	OR	95% CI	Sig.	AOR	95% CI	Sig.
CM	18.3	0.13	(0.10, 0.15)	^ ∗∗∗^	0.23	(0.18, 0.30)	^ ∗∗∗^
Resp.	7.2	0.04	(0.03, 0.07)	^ ∗∗∗^	0.15	(0.09, 0.24)	^ ∗∗∗^
Musc.	7.9	0.05	(0.04, 0.06)	^ ∗∗∗^	0.09	(0.06, 0.13)	^ ∗∗∗^
CM and Musc.	41.2	0.39	(0.32, 0.48)	^ ∗∗∗^	0.45	(0.35, 0.57)	^ ∗∗∗^
CM and Resp.	41.8	0.40	(0.30, 0.55)	^ ∗∗∗^	0.53	(0.38, 0.75)	^ ∗∗∗^
Musc. and Resp.	28.3	0.22	(0.15, 0.32)	^ ∗∗∗^	0.32	(0.21, 0.48)	^ ∗∗∗^
CM, Resp. and Musc.	64.1	(Reference group)					

CM	**18.3**	(Reference group)					
Musc.	7.9	0.38	(0.31, 0.48)	^ ∗∗∗^	0.38	(0.30, 0.49)	^ ∗∗∗^
Resp.	7.2	0.35	(0.24, 0.52)	^ ∗∗∗^	0.62	(0.40, 0.96)	^ ∗^

Musc.	**7.9**	(Reference group)					
Resp.	7.2	0.91	(0.59, 1.39)		1.62	(1.01, 2.60)	^ ∗^

CM and Musc..	**41.2**	(Reference group)					
CM and Resp	41.8	1.03	(0.80, 1.32)		1.19	(0.92, 1.54)	

Musc. and Resp.	**28.3**	(Reference group)					
CM and Musc.	41.2	1.77	(1.29, 2.42)	^ ∗∗∗^	1.42	(1.00, 2.01)	
CM and Resp.	41.8	1.82	(1.25, 2.65)	^ ∗∗^	1.68	(1.11, 2.56)	^ ∗^

Note: Based on 10,528 adults alive at the end of 2009, reported having at least one chronic condition (cardio-metabolic, musculoskeletal, or respiratory condition). All analyses accounted for complex survey design of the Medical Expenditure Panel Survey. The regressions also include intercept terms and parameter estimates for other variables controlled are not presented. Asterisks represent statistically significant group differences compared to the reference group. “0 to 5 drugs” is the reference group for the dependent variable. CM: cardio-metabolic; Musc.: musculoskeletal; Resp.: respiratory; Wt: weighted; OR: Odds ratio; AOR: adjusted Odds ratio; CI: confidence interval.

***P < .001; **0.001 ≤ *P* < 0.01; *0.01 ≤ *P* < 0.05.

## References

[1] Van den Akker M, Buntinx F, Knottnerus JA (1996). Comorbidity or multimorbidity: what’s in a name?. *European Journal of General Practice*.

[2] Fortin M, Soubhi H, Hudon C, Bayliss EA, Van den Akker M (2007). Multimorbidity’s many challenges. *British Medical Journal*.

[3] Van den Akker M, Buntix F, Metsemakers JFM, Roos S, Knottnerus JA (1998). Multimorbidity in general practice: prevalence, incidence, and determinants of co-occurring chronic and recurrent diseases. *Journal of Clinical Epidemiology*.

[4] Fortin M, Bravo G, Hudon C, Vanasse A, Lapointe L (2005). Prevalence of multimorbidity among adults seen in family practice. *Annals of Family Medicine*.

[5] Schneider KM, O’Donnell BE, Dean D (2009). Prevalence of multiple chronic conditions in the United States’ Medicare population. *Health and Quality of Life Outcomes*.

[6] Fortin M, Lapointe L, Hudon C, Vanasse A, Ntetu AL, Maltais D (2004). Multimorbidity and quality of life in primary care: a systematic review. *Health and Quality of Life Outcomes*.

[7] Fortin M, Bravo G, Hudon C (2006). Relationship between multimorbidity and health-related quality of life of patients in primary care. *Quality of Life Research*.

[8] Gijsen R, Hoeymans N, Schellevis FG, Ruwaard D, Satariano WA, Van den Bos GAM (2001). Causes and consequences of comorbidity: a review. *Journal of Clinical Epidemiology*.

[9] Verbrugge LM, Lepkowski JM, Imanaka Y (1989). Comorbidity and its impact on disability. *Milbank Quarterly*.

[10] Marengoni A, von Strauss E, Rizzuto D, Winblad B, Fratiglioni L (2009). The impact of chronic multimorbidity and disability on functional decline and survival in elderly persons. A community-based, longitudinal study. *Journal of Internal Medicine*.

[11] Condelius A, Edberg AK, Jakobsson U, Hallberg IR (2008). Hospital admissions among people 65+ related to multimorbidity, municipal and outpatient care. *Archives of Gerontology and Geriatrics*.

[12] Lee TA, Shields AE, Vogeli C (2007). Mortality rate in veterans with multiple chronic conditions. *Journal of General Internal Medicine*.

[13] Taylor AW, Price K, Gill TK (2010). Multimorbidity—not just an older person’s issue. Results from an Australian biomedical study. *BMC Public Health*.

[14] Lehnert T, Heider D, Leicht H (2011). Review: health care utilization and costs of elderly persons with multiple chronic conditions. *Medical Care Research and Review*.

[15] Safran DG, Neuman P, Schoen C (2005). Prescription drug coverage and seniors: findings from a 2003 national survey. *Health Affairs*.

[16] Wehling M (2009). Multimorbidity and polypharmacy: how to reduce the harmful drug load and yet add needed drugs in the elderly? Proposal of a new drug classification: fit for the aged: letters to the editor. *Journal of the American Geriatrics Society*.

[17] Nolan L, O’Malley K (1988). Prescribing for the elderly. Part I: sensitivity of the elderly to adverse drug reactions. *Journal of the American Geriatrics Society*.

[18] Ruiz B, García M, Aguirre U, Aguirre C (2008). Factors predicting hospital readmissions related to adverse drug reactions. *European Journal of Clinical Pharmacology*.

[19] Fulton MM, Allen ER (2005). Polypharmacy in the elderly: a literature review. *Journal of the American Academy of Nurse Practitioners*.

[20] Nobili A, Marengoni A, Tettamanti M (2011). Association between clusters of diseases and polypharmacy in hospitalized elderly patients: results from the REPOSI study. *European Journal of Internal Medicine*.

[21] http://www.meps.ahrq.gov/mepsweb/data_stats/download_data/pufs/h129/h129doc.pdf.

[22] MacHlin S, Cohen J, Elixhauser A, Beauregard K, Steiner C (2009). Sensitivity of household reported medical conditions in the medical expenditure panel survey. *Medical Care*.

[23] Bushardt RL, Massey EB, Simpson TW, Ariail JC, Simpson KN (2008). Polypharmacy: misleading, but manageable. *Clinical Interventions in Aging*.

[24] Fortin M, Dubois MF, Hudon C, Soubhi H, Almirall J (2007). Multimorbidity and quality of life: a closer look. *Health and Quality of Life Outcomes*.

[25] Meduru P, Helmer D, Rajan M, Tseng CL, Pogach L, Sambamoorthi U (2007). Chronic illness with complexity: implications for performance measurement of optimal glycemic control. *Journal of General Internal Medicine*.

[26] Roehrig C, Miller G, Lake C, Bryant J (2009). Trends: national health spending by medical condition, 1996–2005. *Health Affairs*.

[27] Aparasu RR, Mort JR, Brandt H (2005). Polypharmacy trends in office visits by the elderly in the United States, 1990 and 2000. *Research in Social and Administrative Pharmacy*.

[28] Jörgensen T, Johansson S, Kennerfalk A, Wallander MA, Svärdsudd WK (2001). Prescription drug use, diagnoses, and healthcare utilization among the elderly. *Annals of Pharmacotherapy*.

[29] Linjakumpu T, Hartikainen S, Klaukka T, Veijola J, Kivelä SL, Isoaho R (2002). Use of medications and polypharmacy are increasing among the elderly. *Journal of Clinical Epidemiology*.

[30] Bedell SE, Jabbour S, Goldberg R (2000). Discrepancies in the use of medications their extent and predictors in an outpatient practice. *Archives of Internal Medicine*.

[31] Franssen FM, Spruit MA, Wouters EFM (2011). Determinants of polypharmacy and compliance with GOLD guidelines in patients with chronic obstructive pulmonary disease. *International Journal of COPD*.

[32] Wehling M (2010). Multimorbidity and polypharmacy: which betablocker to use in relation to the pharmacokinetic profile and interaction potential. *Arzneimittel-Forschung/Drug Research*.

[33] Larsen P, Martin JH (1999). Polypharmacy and elderly patients. *Association of Operating Room Nurses Journal*.

[34] Walsh EK, Cussen K (2010). Take Ten Minutes: a dedicated ten minute medication review reduces polypharmacy in the elderly. *Irish Medical Journal*.

[35] Starfield B, Lemke KW, Bernhardt T, Foldes SS, Forrest CB, Weiner JP (2003). Comorbidity: implications for the importance of primary care in “case” management. *The Annals of Family Medicine*.

[36] Prados-Torres A, Poblador-Plou B, Calderón-Larrañaga A (2012). Multimorbidity patterns in primary care: interactions among chronic diseases using factor analysis. *PLoS ONE*.

[37] Coleman EA, Smith JD, Raha D, Min SJ (2005). Posthospital medication discrepancies: prevalence and contributing factors. *Archives of Internal Medicine*.

[38] Poon EJ Medication reconciliation: whose job is it?. *The Online Journal & Forum on Patient Safety and Health Care Quality*.

[39] Fortin M, Stewart M, Poitras M, Almirall J, Maddocks H (2012). A systematic review of prevalence studies on multimorbidity: toward a more uniform methodology. *Annals of Family Medicine*.

